# Histopathological and Transcriptomic Alterations in Metabolic Organs: A Case Study from a Spontaneously Diabetic Rhesus Macaque

**DOI:** 10.3390/ijms27177794

**Published:** 2026-08-31

**Authors:** Qi Yu, Rui Wang, Yuchen Xie, Xu Liu, Wen Ma, Qinghua Liu, Jing Li

**Affiliations:** 1 Key Laboratory of Bio-Resources and Eco-Environment (Ministry of Education), College of Life Sciences, Sichuan University, Chengdu 610065, China; 2 Animal Experimental Center, West China Hospital, Sichuan University, Chengdu 610041, China

**Keywords:** diabetes mellitus, rhesus macaques, metabolic organs, histopathological analysis, transcriptomic analysis

## Abstract

The pathological assessment of diabetes mellitus (DM) in humans is frequently confounded by glucose-lowering therapies. Untreated spontaneously diabetic rhesus macaques (*Macaca mulatta*) provide a valuable model for elucidating the authentic pathology of the disease. In this study, we performed histopathological, immunohistochemical, and transcriptomic analyses on the pancreas, liver, and skeletal muscle of a spontaneous diabetic macaque alongside healthy controls. The results demonstrate that the pancreas exhibited the most severe histopathological damage, with extensive acinar destruction, apoptosis, and prominent IL-17-mediated inflammation, underpinned by activation of the p53 signaling pathway and suppression of exocrine secretion genes. In the liver, damage is driven primarily by innate immune activation and metabolic inflammation, manifesting as metabolic dysfunction-associated steatotic liver disease (MASLD)-like steatotic lesions and insulin resistance-related cytokine expression. In skeletal muscle, endoplasmic reticulum stress emerges as a key molecular event linked to muscle fiber atrophy. Cross-species comparison with human T2DM pancreatic data further identified 20 conserved differentially expressed genes (DEGs) involved in insulin/IGF signaling and chronic inflammation. Collectively, these findings provide a multi-organ perspective on diabetes-induced pathological and transcriptional alterations and underscore the translational relevance of the macaque model for human diabetes research.

## 1. Introduction

Diabetes mellitus (DM) is a complex metabolic disorder characterized by chronic hyperglycemia and multisystem complications, posing a major global health burden. In 2021, the global prevalence of diabetes was estimated to be 6.1% (approximately 529 million cases), with type 2 diabetes mellitus (T2DM) accounting for 96% of cases [1]. More recent projections indicate that its prevalence had exceeded 10% by 2021 and could reach 1.31 billion by 2050 [2]. Beyond elevated blood glucose and glycated hemoglobin, DM is pathologically defined by pancreatic β-cell failure, insulin resistance, systemic inflammation, and dysfunction in multiple insulin-target organs [3,4], culminating in systemic metabolic derangements that drive diverse complications [5,6,7]. The pathophysiology of diabetes involves a complex interplay among the pancreas, liver, skeletal muscle, adipose tissue, and central nervous system [8,9,10,11,12,13,14]. Among these, the skeletal muscle, liver, and adipose tissue are the primary insulin-responsive organs that coordinate glucose uptake, utilization, and storage [15]. When insulin secretion is insufficient or target organ sensitivity is impaired, glucose homeostasis is disrupted, leading to chronic hyperglycemia and the onset of DM [16,17]. Elucidating the pathophysiological alterations and underlying molecular mechanisms in these organs is therefore critical for understanding DM pathogenesis. However, most diabetic patients receive long-term glucose-lowering therapies, which inevitably confound the assessment of disease-associated pathological changes.

Animal models offer an indispensable tool for investigating disease mechanisms. Although rodent models are widely used, they exhibit significant pathophysiological differences from humans. For instance, T2DM rodent models lack islet amyloid polypeptide (IAPP) deposition, a hallmark of human T2DM [18]. In contrast, non-human primates such as rhesus macaques can develop spontaneous diabetes, recapitulating key features of human T2DM, including progressive insulin resistance, β-cell dysfunction, and islet amyloid deposition [19,20,21], making them an ideal translational model. However, spontaneous DM is rare in macaques, and they are often resistant to diabetes induction even with prolonged high-fat, high-sugar dietary interventions [22]. In previous work, we identified a cohort of spontaneously diabetic macaques from a large-scale breeding facility and characterized their metabolic and gut microbiota profiles [23,24]. These animals, having never received glucose-lowering treatment, provide a unique opportunity to study the natural pathological progression of diabetes.

In the present study, we obtained pancreatic, hepatic, and skeletal muscle tissues from one such spontaneously diabetic macaque and performed histopathological and transcriptomic analyses to characterize organ-specific pathological alterations and their molecular underpinnings. Additionally, we compared pancreatic gene expression profiles between T2DM patients and the diabetic macaque to evaluate the translational relevance of this non-human primate model and to identify conserved pathogenic pathways shared between species.

## 2. Results

### 2.1. Histopathological Analysis

#### 2.1.1. Histopathological Alterations

**Figure 1:**
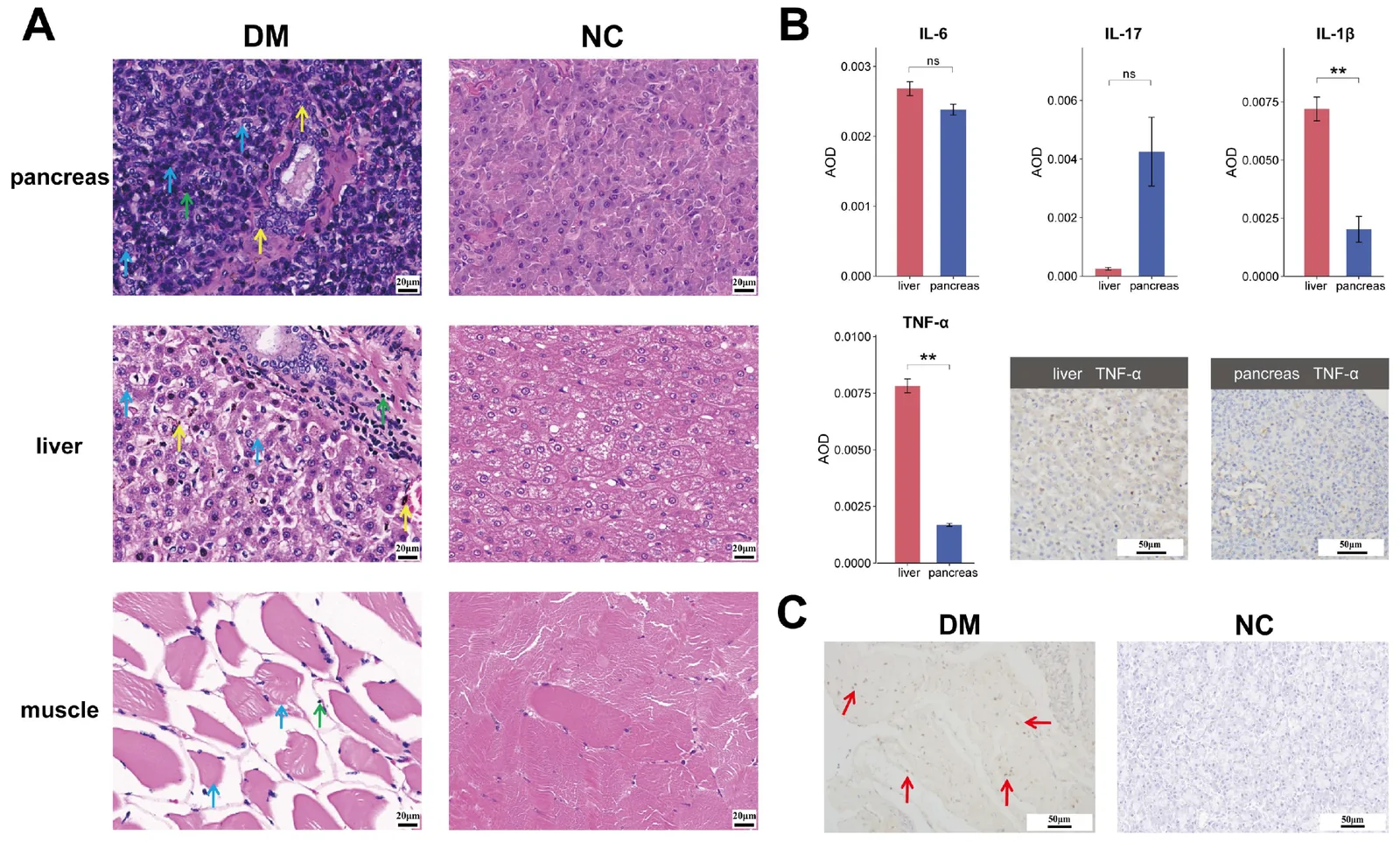
Histopathological analysis and inflammation response in the DM macaque. (**A**) H&E staining of the pancreas, liver, and skeletal muscle (400× magnification; scale bar = 20 μm). Pancreas: Abnormal ductal epithelial hyperplasia (yellow arrows), extensive epithelioid cell proliferation within acini (green arrows), and irregular nuclear size and morphology (blue arrows). Liver: Vacuolar degeneration of hepatocytes (blue arrows), hemosiderin deposition (yellow arrows), and minor lymphocytic infiltration (green arrows). Skeletal muscle: Muscle fiber atrophy (blue arrows) and lymphocytic infiltration (green arrows). (**B**) Average optical density (AOD) of pro-inflammatory cytokines in the liver and pancreas of DM macaque with representative immunohistochemical staining of TNF-α. The AOD values for the control (NC) group were 0.0000 across all tissues. Asterisks indicate statistically significant differences between organs (** *p* < 0.01; independent *t*-test); ns indicates no significant difference (*p* > 0.05). (**C**) TdT-mediated dUTP Nick-End Labeling (TUNEL) staining of pancreatic tissues (200× magnification; scale bar = 50 μm). The AOD value for the DM group was 0.0028 (nuclei showing TUNEL-positive DNA fragmentation signals (red arrows)), compared to 0.0000 in the control group.

H&E staining revealed significant pathological alterations in the pancreas, liver, and skeletal muscle of the diabetic macaque (DM group) compared to controls (Figure 1A). In the pancreas, the acinar structure was largely disrupted and replaced by proliferating epithelioid cells, which exhibited dense, pleomorphic nuclei and an elevated nucleocytoplasmic ratio. The islets of Langerhans were ill-defined, with indistinct borders from the surrounding acini. Additionally, marked ductal epithelial cell proliferation with cytoplasmic basophilia was observed within and between lobules.

The liver displayed metabolic dysfunction-associated steatotic liver disease (MASLD)-like lesions [25], characterized by disorganized lobular architecture and widespread hepatocellular vacuolar degeneration (steatosis). Mild congestion was evident in the central and portal veins, accompanied by prominent hemosiderin deposition within sinusoids and vessels, potentially reflecting diabetes-induced microcirculatory disturbances [26]. Furthermore, bile duct epithelial hyperplasia and sparse lymphocytic infiltration in the surrounding stroma were observed, consistent with chronic inflammation (Figure 1A).

In skeletal muscle, key pathological features included irregular muscle fiber atrophy, resulting in variably sized bundles and sparse fiber arrangement. Some fibers showed marked volume reduction or necrosis, with detachment from the perimysium. Similar to the liver, sparse lymphocytic infiltration was noted in the interstitial spaces (Figure 1A).

#### 2.1.2. Elevated Pro-Inflammatory Cytokine Levels

Given the pronounced pathological and inflammatory changes in the pancreas and liver, immunohistochemistry (IHC) was performed to assess the expression of four pro-inflammatory cytokines (IL-1β, IL-6, IL-17, and TNF-α). While undetectable in control tissues, the average optical density (AOD) values of all four cytokines were markedly elevated in the DM macaque. However, expression levels varied between organs; the liver exhibited significantly higher levels of IL-6, IL-1β, and TNF-α, with IL-1β and TNF-α expression significantly exceeding that in the pancreas (*p* < 0.01, independent *t*-test). Conversely, IL-17 expression was higher in the pancreas than in the liver (Figure 1B).

#### 2.1.3. Increased DNA Fragmentation-Associated Signals in the Pancreas

A TdT-mediated dUTP Nick-End Labeling (TUNEL) assay was performed to evaluate DNA fragmentation-associated signals in the pancreas. Compared to controls, the DM macaque displayed increased numbers of TUNEL-positive cells with golden-brown nuclei (Figure 1C), indicating enhanced DNA fragmentation associated with cellular injury. The AOD value for the DM group was 0.0028, representing increased TUNEL-positive DNA fragmentation signals compared with the control group.

### 2.2. Transcriptomic Analysis and Differentially Expressed Genes

#### 2.2.1. Transcriptome Data

RNA sequencing of the pancreas, liver, and skeletal muscle from diabetic and control macaques generated 49.5 GB of raw data in compressed FASTQ format. Following quality control, an average of 53,069,729 clean reads were obtained per sample (Table 1), with 93.48% successfully aligned to the reference genome (Mmul_10), confirming the high quality and reliability of the dataset for subsequent differential expression analysis.

**Figure 2:**
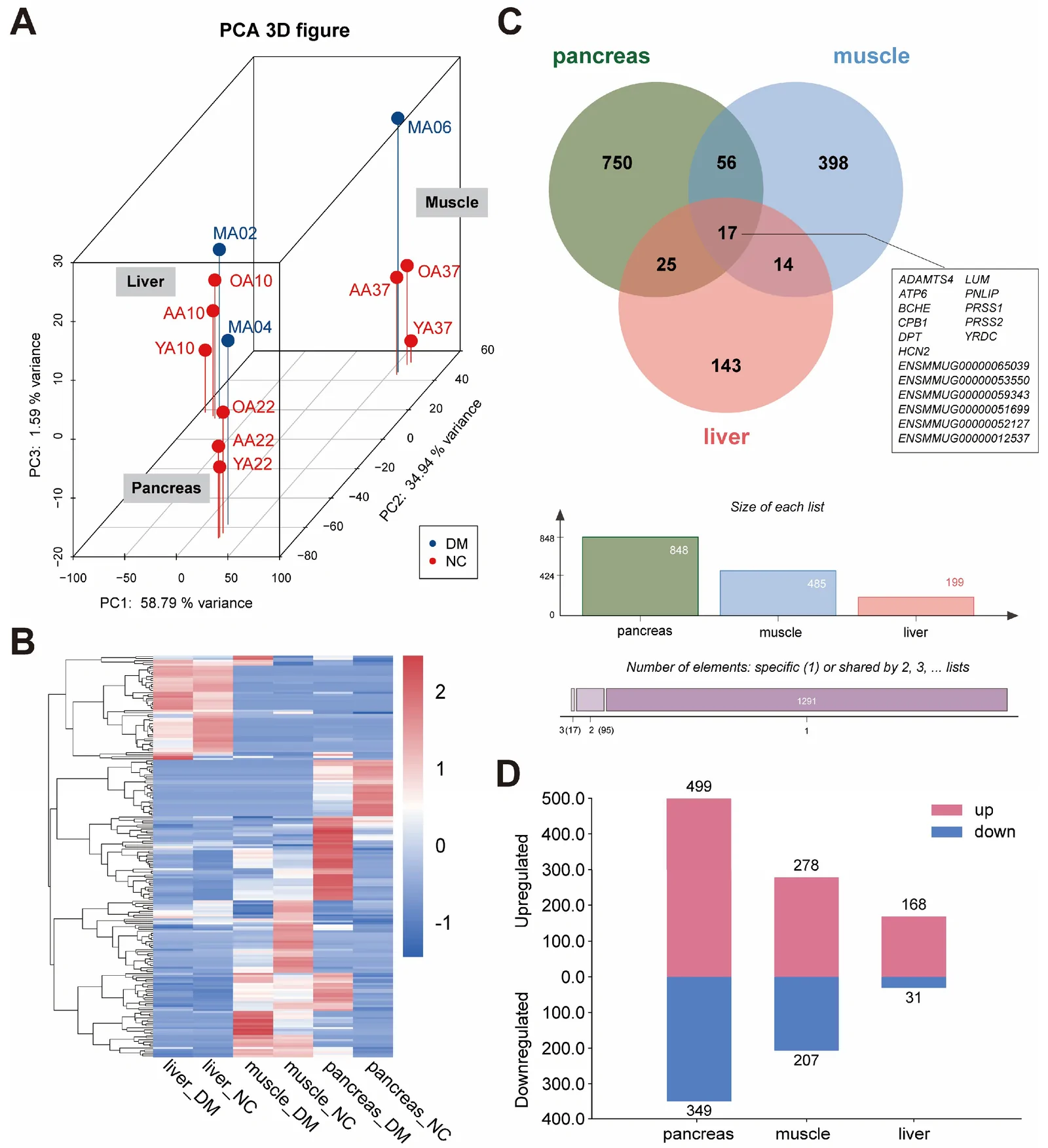
Transcriptomic analysis and differentially expressed genes in three metabolic organs. (**A**) 3D-PCA plot of gene expression profiles across organs. (**B**) Heatmap illustrating the overall gene expression patterns between DM macaque and control macaques. (**C**) Venn diagram showing the overlap of differentially expressed genes (DEGs) among the three organs. Seventeen DEGs were commonly altered across all three tissues. (**D**) Statistical summary of upregulated and downregulated DEGs in the three organs.

Principal component analysis (PCA) revealed that PC1 (58.79%) and PC2 (34.94%) accounted for the majority of variance, distinctly separating the three tissue types (Figure 2A), indicating that organ-specific transcriptional profiles constituted the primary source of variation. Notably, diabetic and control samples were separated along PC3 (1.59%). A hierarchical clustering heatmap corroborated these findings and further demonstrated that, among the three organs, the pancreas exhibited the most pronounced transcriptomic alterations in response to diabetes (Figure 2B).

#### 2.2.2. Shared DEGs Among the Three Organs

Using the criteria |logFC| > 1 and false discovery rate (FDR) < 0.05, a total of 1532 differentially expressed genes (DEGs) were identified between diabetic and control macaques, comprising 945 upregulated and 587 downregulated genes (Table S1). The pancreas exhibited the highest number of DEGs (848), followed by the skeletal muscle (485) and liver (199), suggesting a more pronounced transcriptional response in the pancreatic tissue (Figure 2C,D). Venn diagram analysis revealed that 84.2% (1291/1532) of DEGs were organ-specific, of which 750 (58%) were exclusive to the pancreas. Only 17 DEGs were commonly altered across all three tissues, with 11 of these assigned known gene symbols (Figure 2C).

**Figure 3:**
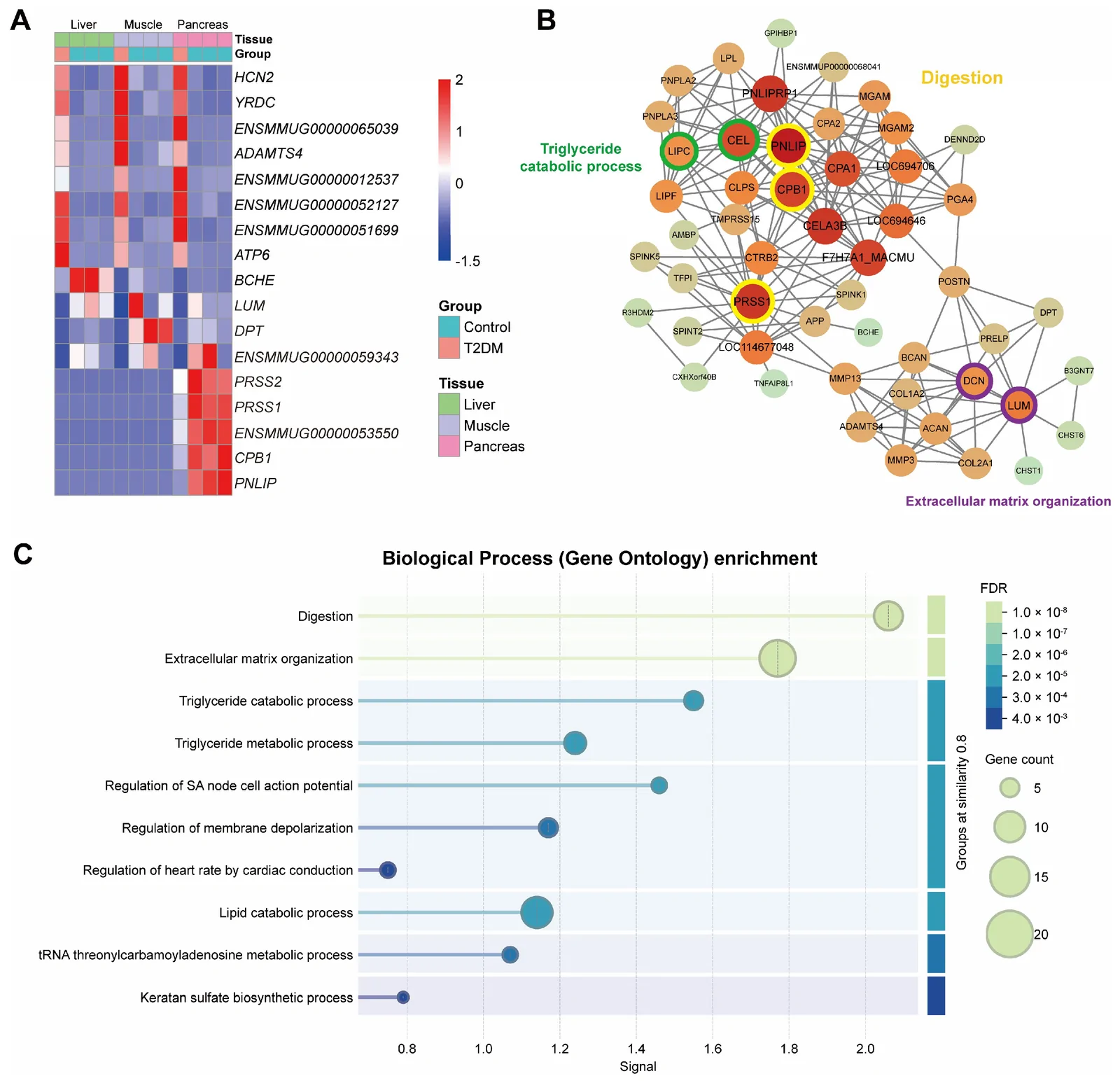
Analysis of the 17 shared DEGs across the pancreas, liver, and skeletal muscle of the DM macaque. (**A**) Heatmap illustrating the expression patterns of the 17 shared DEGs. (**B**) Protein–protein interaction (PPI) network of the 17 shared DEGs. Node colors ranging from green to red represent the degree from low to high. The circles mark key proteins within the Gene Ontology terms (GO-terms) “Digestion” (yellow circles), “Triglyceride catabolic process” (green circles), and “Extracellular matrix organization” (purple circles). (**C**) GO enrichment analysis of the 17 shared DEGs.

The expression patterns of these 17 shared DEGs are presented in the heatmap (Figure 3A). Upregulated genes included *ADAMTS4*, *YRDC*, *HCN2*, *ATP6*, and four unannotated genes, whereas downregulated genes included *LUM*, *DPT*, *BCHE*, and one unannotated gene. Additionally, genes encoding pancreatic exocrine enzymes, *PRSS1*, *PRSS2*, *PNLIP*, and *CPB1*, were also identified as shared DEGs. Although their basal expression levels were lower in the liver and skeletal muscle than in the pancreas, all four were significantly downregulated in the diabetic macaque across all three tissues.

A protein–protein interaction (PPI) network constructed for the 17 shared DEGs revealed highly interconnected modules (Figure 3B). The digestive enzyme cluster centered on PNLIP and PRSS1 displayed the highest connectivity, which was strongly supported by Gene Ontology (GO) enrichment analysis, with the term “digestion” being the most significantly enriched (Figure 3C). Additionally, the enriched terms “Triglyceride catabolic process” and “Triglyceride metabolic process” correspond to the lipid metabolism module centered on LIPC (lipase C hepatic type)/CEL (carboxyl ester lipase) in the PPI network, and the enriched term “Extracellular matrix organization” corresponds to the extracellular matrix (ECM) module centered on LUM and DCN (decorin) in the PPI network. Collectively, these findings indicate that diabetes may exert systemic pathophysiological effects through the synergistic modulation of lipid metabolism, digestive function, and extracellular matrix remodeling.

#### 2.2.3. Organ-Specific DEGs and Functional Enrichment

**Figure 4:**
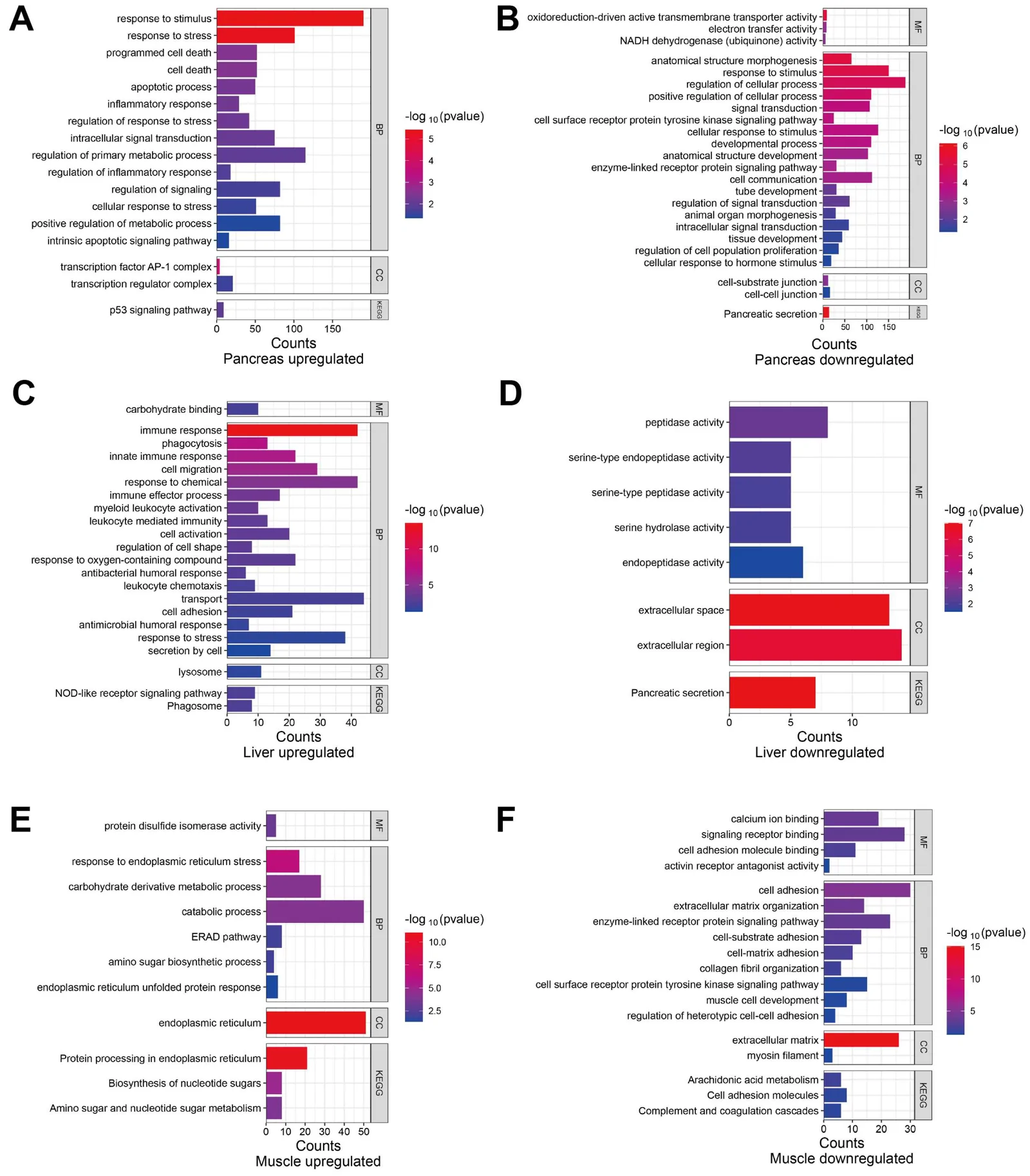
GO and Kyoto Encyclopedia of Genes and Genomes (KEGG) enrichment analysis of upregulated and downregulated DEGs in three organs. (**A**,**B**) Enrichment results of upregulated and downregulated genes in the pancreas. (**C**,**D**) Enrichment results of upregulated and downregulated genes in the liver. (**E**,**F**) Enrichment results of upregulated and downregulated genes in the skeletal muscle.

In the pancreas, upregulated DEGs were significantly enriched in processes related to cellular stress and apoptosis-associated pathways, including “response to stimulus,” “programmed cell death,” and “inflammatory response” (Figure 4A). Kyoto Encyclopedia of Genes and Genomes (KEGG) analysis further implicated the p53 signaling pathway, consistent with the activation of apoptosis-associated molecular pathways observed in transcriptomic analysis and increased DNA fragmentation signals detected by TUNEL staining. Conversely, downregulated DEGs were predominantly enriched in the “pancreatic secretion” pathway (Figure 4B), indicating marked suppression of exocrine function. Collectively, these transcriptional alterations are consistent with the pancreatic structural disruption, inflammatory activation, and impaired exocrine function observed in the pathological analysis.

In the liver, upregulated DEGs were mainly associated with immune and inflammatory responses, with “immune response” being the most significantly enriched term (Figure 4C). Genes involved in innate immunity, phagocytosis, and cell migration were substantially upregulated, corroborating the inflammatory infiltration and hemosiderin deposition observed histopathologically. Enrichment of terms such as “response to chemical” and “response to oxygen-containing compound” further pointed to oxidative stress. KEGG analysis confirmed activation of the phagosome and NOD-like receptor signaling pathways, supporting a role for chronic inflammation and macrophage activity. In contrast, downregulated DEGs were enriched in proteolysis-related processes, including serine hydrolase and endopeptidase activities, as well as extracellular region components (Figure 4D). The downregulation of genes linked to pancreatic secretion was also observed, suggesting reduced protease activity and potential extracellular matrix remodeling.

In skeletal muscle, upregulated DEGs were significantly associated with endoplasmic reticulum (ER) stress, as reflected by enrichment in “response to ER stress,” “ER lumen,” and the KEGG pathway “Protein processing in ER” (Figure 4E). This indicates diabetes-induced disruption of protein homeostasis. Downregulated DEGs were primarily enriched in extracellular matrix organization and cell adhesion terms (Figure 4F), implying structural impairment. These molecular alterations are in line with the muscle fiber atrophy and disorganized arrangement observed in histopathological analysis, linking ER stress and ECM dysregulation to the muscular pathology.

### 2.3. Formatting of Mathematical Components

To identify evolutionarily conserved diabetes-associated pathways or genes, we compared pancreatic transcriptomic profiles from diabetic macaques with a public human dataset (GSE164416) comprising pancreatic samples from T2DM and healthy individuals. In humans, we identified 505 DEGs (460 upregulated, 47 downregulated). Corresponding macaque DEGs were mapped to their human orthologs for cross-species comparison.

A total of 20 shared DEGs with consistent expression patterns were identified between the two species, including 17 upregulated and 3 downregulated genes (Table 2). These genes were primarily involved in the insulin/IGF signaling pathway (e.g., *IGFBP4*, *PAPPA*, *NNMT*) as well as chronic inflammation and immune responses (e.g., *C3*, *IL4R*, *FGB*, *MMP19*), suggesting that diabetes-induced pathological alterations in both primates are closely linked to dysregulation of these metabolic and inflammatory pathways.

**Figure 5:**
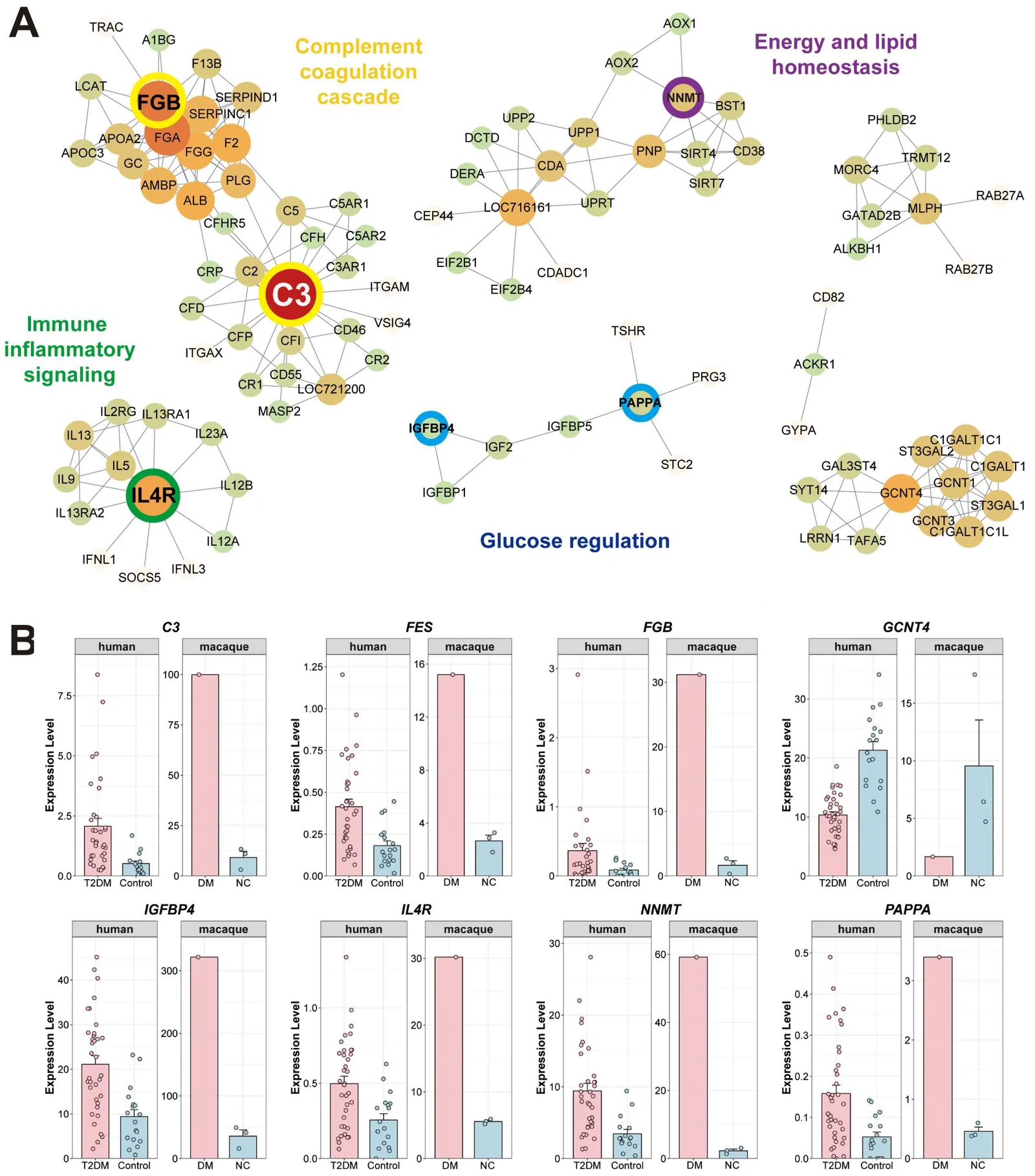
PPI network and expression validation of several shared DEGs. (**A**) PPI network of the 20 shared DEGs. The circles mark key proteins within the modules “Complement-coagulation cascade “ (yellow circles), “Immune-inflammatory signaling” (green circles), “Energy and lipid homeostasis” (purple circles), and “Glucose regulation” (blue circles). (**B**) The expression levels of several shared DEGs in the pancreas of T2DM humans and the DM macaque.

PPI network analysis of the 20 shared DEGs revealed several functional subnetworks (Figure 5A). Within the complement-coagulation cascade module, *C3* and *FGB* emerged as central hubs, while *IL4R* served as a node in the immune-inflammatory signaling cluster, all of which were significantly upregulated in both species (Figure 5B). Additionally, metabolic regulatory modules were identified, including one centered on *NNMT*, involved in energy and lipid homeostasis, and another related to the IGF axis (*IGFBP4* and *PAPPA*), implicated in glucose regulation. Other modules comprised *GCNT4*, *MLPH*, *ACKR1*, *TSPAN15*, and *CAPN13*, with all hub genes upregulated except *GCNT4*, which showed significant downregulation in both diabetic humans and macaques (Figure 5B). These immune-inflammatory and metabolic modules point to complex cross-regulation between metabolic disturbances and islet dysfunction, representing a conserved pathophysiological feature of diabetes in both species.

## 3. Discussion

Diabetes induces multi-organ damage, yet in humans, such pathological consequences are often confounded by glucose-lowering therapies [27,28]. Thus, investigating untreated diabetic animals is essential for developing a comprehensive understanding of the disease [29]. Rhesus macaques share high physiological and pathological similarity with humans, and spontaneous diabetic macaques free of anti-diabetic medication serve as reliable models for studying the natural progression of diabetic pathology. In this study, combined histopathological and transcriptomic analyses of a spontaneous diabetic macaque revealed extensive damage across multiple metabolic organs and elucidated the underlying molecular mechanisms.

The pancreas, liver, and skeletal muscle are recognized as primary targets of diabetic injury [15,30]. In line with this, we observed significant histopathological alterations in all three organs. Notably, 17 DEGs were commonly altered across the three tissues, implicating them as key diabetes-responsive targets. These shared DEGs were enriched in pathways related to digestion, triglyceride catabolism, and ECM organization, suggesting that diabetes-induced pancreatic secretory dysfunction and metabolic disturbances exert systemic effects. PPI network analysis further highlighted pancreatic lipase and trypsinogen as central hubs, reinforcing the widespread impact of diabetes on digestive function, lipid metabolism, and ECM remodeling. These shared genes may serve as potential biomarkers for assessing multi-organ diabetic pathology.

Among the three organs, the pancreas exhibited the most pronounced alterations, characterized by extensive destruction of acinar and islet architecture, accompanied by increased DNA fragmentation signals detected by TUNEL staining and activation of apoptosis-associated pathways (Figure 1). Concurrently, a robust inflammatory microenvironment was observed, with elevated levels of four pro-inflammatory cytokines, particularly IL-17. Given that IL-17 is a key effector of Th17 cells, its elevation suggests T-cell-mediated immunity may contribute to islet injury [31]. Transcriptomic analysis further supported these findings: the pancreas showed the highest number of DEGs, with upregulated genes enriched in the p53 signaling pathway, programmed cell death-related processes, and inflammatory responses, suggesting activation of apoptosis-associated mechanisms during pancreatic injury. Conversely, downregulated DEGs were significantly enriched in the pancreatic secretion pathway, including *PRSS1*, *PRSS2*, *PNLIP*, and *CPB1*, indicating impaired exocrine function that may compromise protein and lipid digestion. Furthermore, we identified pancreas-specific DEGs directly linked to insulin secretion and resistance, such as *SNAP25*, *CEBPB*, *TRIB3*, and *PTPRN2* [32,33,34,35]. Collectively, these results suggest that cytokine-mediated inflammation and Th17-related immune responses activate apoptotic cascades, leading to extensive secretory cell damage in the diabetic pancreas. To definitively characterize the specific cell death pathways contributing to tissue degeneration in diabetic macaque models, future investigations must incorporate specific apoptotic markers, such as cleaved caspase-3 expression and the BAX/BCL-2 ratio.

In the liver, damage was primarily driven by innate immunity and metabolic stress, presenting as MASLD-like pathological features, including steatosis, lymphocytic infiltration, and hemosiderin deposition, which are indicative of chronic inflammatory changes [36,37]. Immunohistochemistry revealed high expression of TNF-α, IL-6, and IL-1β, whereas IL-17 levels were substantially lower than in the pancreas, indicating organ-specific inflammatory mechanisms. Elevated TNF-α and IL-6, well-known inducers of insulin resistance [38,39,40], confirm hepatic insulin resistance in the diabetic macaque, consistent with findings in human T2DM [41,42,43,44]. Transcriptomic analysis supported this, showing upregulation of innate immune pathways, particularly the NOD-like receptor signaling pathway, which can promote IL-1β production [45]. Additionally, enhanced phagocytosis-related gene expression likely underlies the macrophage-mediated erythrocyte clearance and subsequent hemosiderin deposition observed histologically. In skeletal muscle, the primary pathological feature was muscle fiber atrophy, which was associated with severe ER stress at the molecular level. DEGs in muscle were significantly enriched in the “Protein processing in ER” pathway, supporting the established link between ER stress and insulin resistance-induced muscle atrophy [10].

Finally, cross-species comparison between the diabetic macaque and T2DM human revealed 20 shared DEGs with conserved expression patterns in the pancreas, 17 upregulated and 3 downregulated. Among these, upregulated *IGFBP4* and *PAPPA* are negative regulators of the insulin/IGF signaling axis and have been linked to insulin resistance [46,47,48]. The upregulation of complement component *C3* further underscores the central role of inflammation in diabetic pathology [49,50]. These conserved changes suggest that complement activation, inflammatory responses, and metabolic dysregulation are common features of diabetic pancreatic damage in both primates, reinforcing the translational value of the non-human primate model for studying diabetes pathophysiology. Comprehensive multi-tissue transcriptomic comparisons between humans and macaques will provide profound insights into the systemic pathophysiology of T2DM.

## 4. Materials and Methods

### 4.1. Spontaneous DM Macaque and Control Macaques

One spontaneous DM macaque and three control macaques were sourced from Greenhouse Biotechnology Co., Ltd. (Meishan, China; Production License No. SCXK (Chuan) 2019-013). The animal study was approved by the Committee on Ethics of Animal Experiments of the College of Life Sciences, Sichuan University (SCU210429001 and SCU230810001). The study was conducted in accordance with the local legislation and institutional requirements.

The spontaneous DM macaque was an eight-year-old female. The individual exhibited an averaged fasting plasma glucose (FPG) of 7.26 mmol/L in three consecutive measurements at one-month intervals [24], confirming hyperglycemia (Table 3). In contrast, the three control macaques, with an average age of 9 years, consistently showed FPG levels ≤5.0 mmol/L across all measurements. All macaques were housed in continuous full-contact groups, with a separate cage of approximately 25 m^2^ equipped with a standardized diet (50% corn, 14% wheat, 15% soybean meal, 5% wheat bran, 4.5% fish meal, 5.5% sucrose, 5% bone meal, 0.1% multivitamins, 0.1% vitamin C powder, 0.1% trace elements, 0.1% lysine, 0.2% methionine, and 0.1% vitamin B complex powder) and environmental controls, following animal welfare guidelines throughout the sample collection process. None of the macaques had received any medical treatment in the three months before the study.

The macaques were euthanized for sampling. Immediately following euthanasia, necropsies were performed by professional veterinarians. Samples of three metabolic organs (liver, pancreas, and skeletal muscle) were collected. Specifically, tissue fragments (approximately 5 mm^3^ in size) were used for histopathological analysis. For transcriptomic sequencing, the tissues were rinsed with RNase-free phosphate-buffered saline (PBS), promptly snap-frozen in liquid nitrogen, and subsequently stored at −80 °C. Fresh tissue specimens for histopathological analysis were fixed in 4% paraformaldehyde for a minimum of 24 h.

### 4.2. Histopathological Analysis

#### 4.2.1. Hematoxylin and Eosin Staining

Fixed specimens were trimmed, dehydrated through graded ethanol (75–100%), and embedded in paraffin. Sections (3 μm in thickness) were cut, floated at 40 °C, and dried at 60 °C. Following deparaffinization and rehydration, sections were stained with hematoxylin, differentiated, and blued, followed by eosin counterstaining for 5 min. After final dehydration and clearing, the slides were mounted with neutral resin. Histopathological changes were examined using a Nikon Eclipse E100 microscope and a DS-U3 imaging system (Nikon Corporation, Tokyo, Japan).

#### 4.2.2. Immunohistochemistry

The expression of four kinds of pro-inflammatory cytokines (IL-1β, IL-6, IL-17, and TNF-α) was detected via IHC. Paraffin-embedded sections (3 μm) were deparaffinized, rehydrated, and subjected to heat-induced antigen retrieval. Following the blocking of endogenous peroxidase and non-specific binding (5% normal goat serum), sections were incubated overnight at 4 °C with primary antibodies against IL-1β (1:400), IL-6 (1:100), IL-17 (1:800), and TNF-α (1:400) (Servicebio, Wuhan, China). Subsequently, the sections were incubated with an HRP-conjugated secondary antibody (1:500; Biosharp, Beijing, China) for 50 min. Immunoreactivity was visualized with DAB, and nuclei were counterstained with hematoxylin. For quantitative analysis, three random fields per section were captured at 200× magnification using a consistent background light. The integrated optical density (IOD) and the positive area (AREA) of the brownish-yellow signals were measured using Image-Pro Plus 6.0 (Media Cybernetics, Inc., Rockville, MD, USA). The average optical density (AOD = IOD/AREA) was then calculated to evaluate the expression levels of these cytokines [51].

#### 4.2.3. TUNEL Apoptosis Assay

The TUNEL assay was employed to evaluate cell apoptosis in pancreatic tissues. Deparaffinized sections were treated with 20 μg/mL Proteinase K (37 °C, 20 min) and 0.5% Triton X-100 (20 min). After washing, endogenous peroxidase activity was blocked with 3% H_2_O_2_ for 25 min in the dark. According to the manufacturer’s instructions (DAB TUNEL Kit, Servicebio, Wuhan, China, G1507), sections were sequentially incubated with Equilibration Buffer (10 min), TdT incubation buffer (37 °C, 1–2 h), and Streptavidin-HRP reaction solution (37 °C, 30 min). Following color development with DAB (Biosharp, Beijing, China, BL732A), apoptotic nuclei exhibited a brownish-yellow hue, while total nuclei were counterstained with hematoxylin, differentiated, and blued. Finally, sections were dehydrated, cleared, and mounted. Image acquisition and semi-quantitative analysis (AOD = IOD/AREA) were performed using the same methodology as described for IHC.

### 4.3. Transcriptome Sequencing and Data Analysis

#### 4.3.1. Library Preparation and Sequencing

Total RNA was extracted from each organ sample using Trizol reagent (Invitrogen, Carlsbad, CA, USA). The purity, concentration, and integrity of the RNA were assessed using a spectrophotometer and an Agilent 2100 Bioanalyzer (Agilent Technologies, Santa Clara, CA, USA). Only samples meeting the quality control standards were used for library construction. Subsequently, ribosomal RNA was removed using the Ribo-Zero kit, and cDNA libraries were constructed with the NEBNext Ultra Directional RNA Library Prep Kit (NEB, Ipswich, MA, USA). After quality validation, the libraries were sequenced on the Illumina HiSeq X Ten platform using a paired-end (PE150) strategy. This process generated 150 bp paired-end reads for further bioinformatic analysis.

#### 4.3.2. Data Analysis

Raw reads were first processed using the NGS QC Toolkit to perform quality control. Low-quality reads and Illumina sequencing adapters were removed to obtain high-quality clean reads. These clean reads were aligned to the rhesus macaque reference genome (Mmul_10) using HISAT2 [52,53]. The resulting SAM files were converted to BAM format using SAMtools v1.19.2 [54]. Subsequently, gene and transcript quantification were performed using featureCounts v2.0.6 to generate a raw read count matrix [55]. Genes with low expression levels across all samples were removed. Gene expression levels were normalized as Fragments Per Kilobase of exon model per Million mapped fragments (FPKM).

#### 4.3.3. Differential Expression and Functional Enrichment Analysis

Differential expression analysis was performed using the R package edgeR v4.0.3 [56]. DEGs were identified based on the criteria of |log2 FC| ≥ 1 and FDR ≤ 0.05. Functional enrichment analysis of DEGs, including GO and KEGG pathways, was conducted using g:Profiler based on the rhesus macaque reference genome [57]. Data visualization was performed using the R package ggplot2 v4.0.3 [58]. To explore the relationships among DEGs, a protein–protein interaction (PPI) network was constructed using the STRING database (http://string-db.org) with a minimum required interaction score of 0.700 (highest confidence). The PPI network was visualized using Cytoscape v3.8.2 [59].

### 4.4. Cross-Species Transcriptomic Comparative Analysis

To explore similarities in gene expression between the diabetic macaque and diabetic humans, human pancreatic transcriptome data (GSE164416) were downloaded from the NCBI GEO database, comprising 39 T2DM patients and 18 non-diabetic controls [60]. Raw sequencing data were aligned to the human GRCh38 reference genome, and transcript count matrices were generated following the same methodology used for the macaque transcriptome (see Section 4.3). Differential expression analysis was performed using the edgeR package. The transcript IDs (Ensembl IDs) of human pancreatic DEGs were mapped to their corresponding gene symbols using the biomaRt package v2.66.2. Similarly, macaque pancreatic DEGs were mapped to human orthologous gene symbols. These results were compared with the human DEGs to identify shared differentially expressed genes between the diabetic macaque pancreas and the human T2DM pancreas.

## 5. Conclusions

This study characterized the histopathological and transcriptomic alterations in the pancreas, liver, and skeletal muscle of a spontaneous diabetic rhesus macaque, providing a multi-organ perspective on diabetic pathology without glucose-lowering treatment. It demonstrates that the pancreas is the most severely affected organ, with extensive acinar destruction, DNA fragmentation-associated injury, apoptosis-related pathway activation, and prominent IL-17-mediated inflammation, underpinned by activation of the p53 signaling pathway and suppression of exocrine secretion genes. Meanwhile, liver and muscle demonstrate distinct inflammatory and stress-related pathologies. Cross-species comparison with human T2DM pancreatic transcriptomes further revealed 20 conserved differentially expressed genes, highlighting shared pathogenic mechanisms involving insulin/IGF signaling dysregulation and complement-mediated inflammation. This study provides a foundational framework for future investigations in larger cohorts to elucidate the full spectrum of diabetes-induced multi-organ pathology.

## Figures and Tables

**Table 1:** Summary of transcriptome sequencing data.

Group	Sample	Tissue	Raw Reads	Raw Bases	Clean Read Rate (%)	Raw Q30 (%)	Mapped (%)
DM	MA02	liver	48,119,166	7,217,874,900	96.29	92.31	98.59
	MA04	pancreas	47,085,112	7,062,766,800	96.12	92.68	98.69
	MA06	muscle	55,255,744	8,288,631,600	97.60	93.41	98.48
NC	AA10	liver	58,382,962	8,757,444,300	90.76	91.49	93.26
	AA22	pancreas	52,683,724	7,902,558,600	90.41	91.20	94.01
	AA37	muscle	51,429,624	7,714,443,600	90.60	91.42	92.35
	OA10	liver	56,021,928	8,403,289,200	90.72	91.58	91.79
	OA22	pancreas	52,377,584	7,856,637,600	91.18	91.88	93.52
	OA37	muscle	54,305,360	8,145,804,000	96.31	90.59	90.18
	YA10	liver	54,626,670	8,194,000,500	89.56	90.56	90.79
	YA22	pancreas	52,758,908	7,913,836,200	89.52	90.47	91.17
	YA37	muscle	53,789,970	8,068,495,500	90.39	91.21	89.00
Average			53,069,729	7,960,481,900	92.45	91.57	93.49

**Table 2:** Shared DEGs in the pancreas of diabetic macaque and humans.

Shared DEG Gene Symbol		Shared DEG Full Gene Name	logFC (Human)	*p* (Human)	logFC (Macaque)	*p* (Macaque)
up	*IGFBP4*	Insulin-Like Growth Factor Binding Protein 4	1.437	9.83 × 10^−6^	2.196	4.66 × 10^−4^
	*FES*	FES Proto-Oncogene, Tyrosine Kinase	1.620	1.15 × 10^−5^	1.555	1.35 × 10^−3^
	*C3*	Complement C3	2.275	1.82 × 10^−5^	2.473	6.75 × 10^−4^
	*CAPG*	Capping Actin Protein, Gelsolin-Like	1.585	1.01 × 10^−4^	2.081	1.65 × 10^−6^
	*TYMP*	Thymidine Phosphorylase	1.544	1.30 × 10^−4^	2.362	1.31 × 10^−4^
	*MLPH*	Melanophilin	1.314	1.48 × 10^−4^	3.283	2.10 × 10^−4^
	*AKNA*	AT-Hook Transcription Factor	1.218	1.97 × 10^−4^	1.801	6.62 × 10^−5^
	*NNMT*	Nicotinamide N-Methyltransferase	1.298	3.27 × 10^−4^	3.726	1.87 × 10^−13^
	*TSPAN15*	Tetraspanin 15	1.241	4.48 × 10^−4^	1.755	1.39 × 10^−4^
	*MMP19*	Matrix Metallopeptidase 19	1.715	4.65 × 10^−4^	1.962	6.57 × 10^−4^
	*IL4R*	Interleukin 4 Receptor	1.167	5.33 × 10^−4^	1.478	1.15 × 10^−4^
	*ADAMTS8*	ADAM Metallopeptidase With Thrombospondin Type 1 Motif 8	1.631	1.51 × 10^−3^	2.524	1.27 × 10^−3^
	*ACKR1*	Atypical Chemokine Receptor 1 (Duffy Blood Group)	2.792	1.69 × 10^−3^	2.319	1.07 × 10^−3^
	*C11orf96*	Chromosome 11 Open Reading Frame 96	1.518	2.83 × 10^−3^	2.843	3.86 × 10^−4^
	*FGB*	Fibrinogen Beta Chain	2.782	3.19 × 10^−3^	3.241	3.46 × 10^−4^
	*PAPPA*	Pappalysin 1	1.253	4.23 × 10^−3^	1.922	3.48 × 10^−4^
	*LAMP3*	Lysosomal-Associated Membrane Protein 3	1.707	4.57 × 10^−3^	4.173	3.14 × 10^−4^
down	*GCNT4*	Glucosaminyl (N-Acetyl) Transferase 4	−1.051	1.19 × 10^−13^	−3.455	2.63 × 10^−4^
	*CAPN13*	Calpain 13	−1.065	3.52 × 10^−8^	−3.168	2.82 × 10^−7^
	*HS6ST2*	Heparan Sulfate 6-O-Sulfotransferase 2	−1.352	3.67 × 10^−7^	−2.863	1.12 × 10^−5^

**Table 3:** Metabolic characteristics and clinical parameters of the diabetic macaque and control macaques.

Group	Sample ID	Sex	FPG (mmol/L)	FPI (mIU/L)	HOMA-IR	HbA1c (%)
DM	MA	F	7.26 ± 0.34	11.93	3.85	4.90
NC	YA	F	4.47 ± 0.49	7.65	1.52	4.00
	AA	F	3.23 ± 0.17	6.05	0.87	4.10
	OA	F	3.18 ± 0.41	5.73	0.81	2.30

Data are presented as mean ± SD for fasting plasma glucose values obtained from repeated measurements. FPG, fasting plasma glucose; FPI, fasting plasma insulin; HOMA-IR, homeostatic model assessment of insulin resistance, calculated using the formula FPI × FPG/22.5.

## Data Availability

The datasets generated for this study can be found in the National Genomics Data Center (NGDC), China National Center for Bioinformation (CNCB), under BioProject accession number PRJCA062918 and GSA accession number CRA041895.

## Supplementary Materials

The following supporting information can be downloaded at: https://www.mdpi.com/article/10.3390/ijms27177794/s1.

## Abbreviations

The following abbreviations are used in this manuscript:

DM	Diabetes mellitus
T2DM	Type 2 diabetes mellitus
IAPP	Islet amyloid polypeptide
MASLD	Metabolic dysfunction-associated steatotic liver disease
TUNEL	TdT-mediated dUTP Nick-End Labeling
IHC	Immunohistochemistry
AOD	Average optical density
FDR	False discovery rate
DEGs	Differentially expressed genes
PPI	Protein–protein interaction
GO	Gene Ontology
ER	Endoplasmic reticulum
KEGG	Kyoto Encyclopedia of Genes and Genomes
ECM	Extracellular matrix
FPG	Fasting plasma glucose
PBS	Phosphate-buffered saline
IOD	Integrated optical density
AREA	Positive area
FPKM	Per million mapped fragments
